# New Insights on Biofilm Antimicrobial Strategies, 2nd Volume

**DOI:** 10.3390/antibiotics11070908

**Published:** 2022-07-07

**Authors:** Andreia S. Azevedo, Luís D. R. Melo

**Affiliations:** 1 LEPABE-Laboratory for Process Engineering, Environment, Biotechnology and Energy, Faculty of Engineering, University of Porto, 4200-465 Porto, Portugal; 2 ALiCE-Associate Laboratory in Chemical Engineering, Faculty of Engineering, University of Porto, 4200-465 Porto, Portugal; 3 i3S-Instituto de Investigação e Inovação em Saúde, University of Porto, 4200-135 Porto, Portugal; 4 IPATIMUP-Instituto de Patologia e Imunologia Molecular, Universidade do Porto, 4200-135 Porto, Portugal; 5 Centre of Biological Engineering (CEB), University of Minho, 4710-057 Braga, Portugal; 6 LABBELS—Associate Laboratory, Braga/Guimarães, Portugal

In biofilms, microorganisms are able to communicate together and assemble by themselves, creating a consortium with different properties from the original free-floating microorganisms. In fact, biofilm cells bind strongly to a living or non-living surface, enclosed in a self-produced extracellular matrix that is composed of extracellular polymeric substances. One benefit of this lifestyle is the increased resistance or tolerance to antimicrobial agents (e.g., antibiotics). Hence, research on the development of alternative strategies to prevent and control biofilms is highly relevant for society in terms of human health, industry and the environment. Different approaches to prevent or control biofilms using antibiotic alternative strategies were submitted to this Special Issue.

An important topic is the study of quorum sensing (QS) mechanisms during biofilm development. Daza et al. evaluated the use of alkylglycerols as QS inhibitors. The effect on the biofilm formation of fifteen natural enantiopure alkylglycerols was evaluated on *Chromobacterium violaceum*. The authors observed a dose-dependent response using each alkylglycerol at subinhibitory concentrations, with a maximum response of 97.2% reduction. (2S)-3-O-(cis-13′-docosenyl)-1,2-propanediol was the best QS inhibitor with just 20 μM [1].

The use of chemical compounds, bacteriophages and enzymes are also alternative approaches with the potential to control the bacterial growth and the biofilm development. For instance, Alfarrayeh et al. evaluated the effect of Caffeic Acid Phenethyl Ester (CAPE) on different *Candida* species. Both caspase-dependent and caspase-independent apoptosis were observed after CAPE treatment. The minimum biofilm inhibitory concentration oscillated between 50 and 100 µg/mL. Regarding biofilm control, the authors observed a low to high capability to control mature *Candida* biofilms [2].

In another study, Pereira et al. used gapmers and steric blockers as antisense oligonucleotides against *Escherichia coli*. These molecules were conjugated with vitamin B12 by copper-free azide-alkyne click-chemistry. The authors observed that despite the strong interaction with *E. coli*, conjugates were mostly located on the outer membrane. Only 6–9% reached the cytosol, which was not sufficient to inhibit bacterial growth. The authors concluded that this low internalization of conjugates was affected by *E. coli*’s low uptake for vitamin B12 and further studies are necessary before applying it to infectious biofilms [3].

Additionally, using stainless steel surfaces, González-Gómez et al. studied the efficacy of novel bacteriophages against *E. coli* biofilms. Three bacteriophages isolated from ground beef and poultry liver samples with a podovirus-like morphology were used alone or in combination to treat biofilms for 2, 14 and 48 h. Some very significant bacterial cell count reductions were observed after treatment, with it being important to note that the treatment success was influenced by the bacterial strain used, the used phage, phage concentration and biofilm formation stage [4].

The effects of enzymatic and chemical treatments were compared to remove mixed-species biofilms on surfaces simulating the food processing environment. Iñiguez-Moreno et al. used a novel chemical product named Sanicip Bio Control or peracetic acid as chemical agents and protease or α-amylase for enzymatic treatments. Biofilms were formed on stainless steel or polypropylene B and different media were used (whole milk, TSB with meat extract and TSB with chicken egg yolk). Despite the success of some treatments, in general the results were strongly affected by food contact surfaces and the surrounding media [5].

A combined treatment is another interesting strategy; Aljaafari et al. investigated the effect of various thermal shocks treatments (different temperature and times), the effect of different ciprofloxacin hydrochloride concentrations and the interaction of antibiotics and thermal shock on *Pseudomonas aeruginosa* biofilms. In addition, to assess the viability after the thermal shock, the biofilms were subsequently re-incubated under the initial conditions. To generate biofilms with different population densities, structures, and maturities, *P. aeruginosa* biofilms were grown on 4-well dishes at 160 rpm in an incubator (ST biofilms) and in a Drip Flow Reactor (DFR biofilms). The authors concluded that the use of ciprofloxacin does not appear to enhance thermal shock directly. However, they suggested that thermal shock and antibiotics act with strictly orthogonal mechanisms on *P. aeruginosa* biofilms, decreasing the intensity of thermal shock needed [6].

Barros et al. tested the antimicrobial activity of biocide benzalkonium chloride (BAC) immobilized in millimetric aluminum oxide particles, against *Escherichia coli* bacteria in the planktonic state. The alumina particles were functionalized by using a nitrogen precursor (Dopamine (DA)). At the highest particle concentration (3000 mg/L), an inactivation of bacterial cells within 5 min was observed. In addition, a total loss of membrane integrity was achieved after 15 min for all tested concentrations. However, when reusing the Al_2_O_3_-DA-BAC particles, a higher contact time was needed to reach total inactivation. The authors concluded that Al_2_O_3_-DA-BAC particles are a suitable antimicrobial agent for the treatment of continuous water systems with minimal environmental and health impacts [7].

Surface engineering approaches are another successful strategy to control biofilms. Sunthar et al. analyzed the antibacterial properties of Cellulose Acetate (CA) reinforced with different weight percentages of aluminum nitride (AlN) composites against *Staphylococcus epidermidis* and *E. coli*. The results showed an effective antibacterial effect when AIN was added in weight percentages >10 wt.%, suggesting the potential application of CA/AlN composites as alternative materials for plastic packaging in the food industry. However, the authors suggest that the degradability and stability of this composite material should be studied in future work [8].

Using a different and innovative approach, Carvalho et al. evaluated the effect of two probiotic strains (*Lactobacillus plantarum* and *Lactobacillus rhamnosus*) in displacing *E. coli* and Staphylococcus aureus pre-formed biofilms. The biofilms were grown under conditions that mimic the urological devices, including silicone surfaces, artificial urine medium and a shear stress similar to those found inside of urinary catheters. This is the first study that demonstrates the ability of *L. plantarum* and *L. rhamnosus* to displace pre-established biofilms of *E. coli* and *S. aureus*. These results showed the potential of these Lactobacillus strains to control the development of biofilms on urinary tract devices [9].

Finally, Esteves et al. provided a review to evaluate the significance of the incorporation of antibacterial coatings in the reduction in the occurrence of bacterial infections and evaluate its effect on dental implant success rate. The authors summarize the diverse strategies proposed to enhance the antibacterial properties of titanium dental implants. This is significant because antibacterial coatings are a promising solution to control and prevent bacterial infections that compromise dental and orthopedic implant success [10].
